# Remote clinical training practice in the neurology internship during the COVID-19 pandemic

**DOI:** 10.1080/10872981.2021.1899642

**Published:** 2021-03-08

**Authors:** Miao He, Xiang-qi Tang, Hai-nan Zhang, Ying-ying Luo, Zhen-chu Tang, Shu-guang Gao

**Affiliations:** a Department of Neurology, The Second Xiangya Hospital, Central South University, Changsha, Hunan, China; b Department of Orthopaedics, Xiangya Hospital, Central South University, Changsha, Hunan, China; c National Clinical Research Center of Geriatric Disorders, Xiangya Hospital, Central South University, Changsha, Hunan, China

**Keywords:** Online clinical training, neurology internship, SPOC, blending learning mode

## Abstract

**Background**: During the current COVID-19 pandemic, offline clinical education was mandated to suspend at the neurology department of many teaching hospitals globally, yet there is insufficient evidence regarding the preferred practice and methods for online neurology intern training course.

**Objective**: The investigation aimed to examine whether the online neurology training course based on Small Private Online Course (SPOC) and blending learning mode can achieve a good effect and cater for interns from different medical programs and whether the learning group size affects the teaching effect.

**Design**: The subjects were 92 students enrolled in the neurology internship at the Second Xiangya Hospital of China from 9 March to 9 August 2020. After completing the online course, the final scores and evaluation results were compared among different groups of interns, and their preference to distinct contents of the course was analyzed. Statistical analysis was performed using the SPSS program (version 22.0).

**Results**: Our online course received consistent positive recognition from the interns. Ninety-nine percent of the interns recommended incorporating the online course into the conventional offline training program after the pandemic. There was no significant difference between interns from different programs concerning the final scores and course evaluation. A smaller learning group size (<15 students) could achieve a better teaching effect than a larger group size (p < 0.05). The interns preferred interactive discussions, and course contents that they can get practice and feedback from, rather than video watching and didactic lectures.

**Conclusions**: The online neurology intern training course based on SPOC and blending learning mode is worthy of popularization in a large student base. The teaching effect of an online intern training program may be improved by limiting the group size to less than 15 students and encouraging more interactive discussion, more practice and feedback.

## Introduction

During the current Coronavirus Disease 2019 (COVID-19) pandemic, medical schools and teaching hospitals in China and in many other countries were mandated to suspend offline clinical training activities as well as student-involved clinical practice [1,2]. Instead, online teaching was implemented as a measure to prevent disease transmission while ensuring the continuity of clinical education [3–5]. However, the existing evidence is insufficient regarding what the preferred practice and methods are for the online neurology intern training course.

Even though the COVID-19 pandemic is not the first outbreak of coronavirus that has an impact on clinical education, the rapidly-developing electrification education based on Internet has made remote teaching more diverse, more innovative and more effective [6–8]. However, the hurried and unprepared switch from the conventional ‘at the bedside’ clinical education to online live classes is still a huge challenge [9,10]. Small Private Online Course (SPOC) refers to a version of distance course that is often used locally among a limited group of students [11,12]. Additionally, teaching based on the blending learning mode (e.g., combination of didactic classes, flipped classroom, and case-based learning) is also becoming popularized in online programs nowadays as a way to optimize learning effectiveness. Specifically, flipped classroom is an active learning model that engages students in the self-directed learning process to improve their understanding and application of the content [13,14]. Through case-based learning, the students can learn by solving real-world problems such as realistic patient care situations. In general, the blending learning mode has been widely employed in medical education [15,16].

The department of neurology receives patients with disorders involving the nervous system. The particularities of neurology intern training are as follows: First, the students are expected to receive more intensive training in clinical reasoning and critical thinking since many neurological disorders present similar clinical features and radiographic findings. Besides, difficult, complicated, undiagnosed or rare clinical cases are common in the neurology department. Second, long-term follow-ups are required and implemented more commonly in neurology than in other disciplines. Third, the training of theoretical knowledge and practical skills is equally important. The offline conventional clinical training plays an essential and irreplaceable role in neurology intern training as effective experience can only be obtained from real clinical settings. However, even before the pandemic, certain shortcomings of the conventional intern training program have been exposed. For example, since highly qualified physicians in China are expected to undertake clinical, teaching and research duties concurrently, the amount of time they can spend on educating interns is very limited. Additionally, due to the short period of time the interns can stay in one department (usually 2–3 weeks), they may miss the opportunities to observe and learn some typical or rare clinical cases [17]. Considering the unique advantages of online learning mode, a quality online clinical training program can possibly overcome, at least in part, the shortcomings of the conventional intern training program and achieve a good teaching effect [18].

Educational challenges faced by medical intern training programs have been widely noted during the pandemic, while remote education has been taken as a typical solution to address them [19,20]. In view of the shortage of relevant experience and literature, this investigation aimed to examine whether the online neurology training course based on SPOC and blending learning mode can achieve a good application effect and cater for interns from different medical educational programs during the pandemic period, as well as whether the learning group size has an impact on the teaching/learning effect, and to analyze how a remote clinical training course can be refined.

## Methods

### Participants

A total of 92 interns from the medical school of Central South University in China, who were rotated in the neurology department at the Second Xiangya Hospital, were engaged in our distance intern training program. The interns were divided into 3 groups based on their educational programs and grades so as to verify whether our online training course caters for students from different programs and with different knowledge sets. Group 1 included 47 students (51%) that were in their 5th year of a 5-year clinical medicine program (for a bachelor of medicine degree). Group 2 included 37 students (40%) that were in their 6th or 7th year of an 8-year clinical medicine program (for a doctor of medicine degree). Group 3 included 8 students (9%) that were in their 5th year of a 5-year psychiatry program. The 3 groups were mixed up and separated into 6 batches, and then attended our online course at different points of time from March 9^th^ to 9 August 2020. Each batch of students was considered a learning group/class. The 6 batches (or learning groups) were composed of 14, 22, 10, 17, 13, and 16 students, respectively (in order of the time they received the training). For gender composition, 40 were female (43%) and 52 were male (57%). All of them had responded to our survey upon the completion of the course. All the participants provided informed consent.

### Course arrangement and the survey

Our program was a 3-week online course focusing on clinical neurology education. All our faculty members were qualified teachers with relevant clinical experience. The training course was based on SPOC and blending learning mode including didactic classes, some elements of flipped classroom, and case-based learning. Tencent Class, which is a cloud-based application program allowing live lectures, videos, PowerPoint, screening sharing, and interactive case discussions to increase student engagement, was used as the live broadcast platform. The curriculum of our classes covered new patients’ admission, videos of typical clinical cases with instructions, videos of difficult clinical case discussion, interactive real-world clinical case discussions, medical record writing, theoretical lectures, practice training, and feedback including physical examination of the nervous system, cardiopulmonary resuscitation (CPR) and lumbar puncture, as well as recommended participation of online conferences and article reading to expand the students’ horizons (Supplemental Table 1). The teaching plan was released at least 1 week ahead of time by the instructors. The training duration per day lasted for about 3 hours in weekdays, with a short break after each hour. To replicate live clinical exposure as far as possible, we captured a mass of clinical videos under real clinical environments after obtaining permission from the patients and their family (with their personal information masked). For interactive clinical case discussions, the patient cases were given to the students prior to the class alongside a list of relevant questions. The students were allowed to discuss the cases in pre-assigned small groups. Then, the instructor would guide interactive discussions in the online class. After-class assignments included practice problems relevant to the content of online classes, medical record writing after watching videos of patient encounter, clinical case analyses, and video recording of home practice. Individual feedback to the assignment would be given within a specified time. The assessment and grading strategy were based on attendance, assignments, practical and theoretical examinations. The final score was graded based on the comprehensive evaluation of attendance, assignments, practical and theoretical examinations. For the practical examination, the interns were required to shoot a video of themselves doing physical examination, CPR and lumbar puncture by using mannequins or their family members as models. The theoretical examination was a 60-minute open-book examination which was implemented online through screen sharing. The examination consisted of 15 single-choice questions (4 points per question) and 2 essay questions (20 points per question) (same as the examination used in our pre-COVID19 offline training curriculum). Both examinations were marked by our faculty in a unified standard. After the schedule was fixed, a WeChat group would be created for each group of interns, with the faculty member in charge of teaching involved. The WeChat group was used to disseminate information such as curriculum contents and class notifications, as well as teaching-related documents such as PowerPoint slides and assignments. The students could also receive correct answers to the questions in a timely manner through the WeChat group. Throughout the investigation, we managed to cooperate with other hospitals to share teaching and clinical resources so as to provide our students with more comprehensive learning materials.

**Table 1.** Survey questions about satisfaction to the course and feedbacks from different groups of students with a 10-point scale (expressed as median score (interquartile range))

Items	Group 1 (N = 47)	Group 2 (N = 37)	Group 3 (N = 8)	*P*-Value^a^
Your satisfaction to the curriculum framework and logic of the course	10(9–10)	10 (9–10)	9.5 (9–10)	0.79
Your satisfaction to the practicality of knowledge you obtained in the course	10(8–10)	10 (9–10)	9 (8–9.75)	0.20
Your satisfaction to the new knowledge and concepts you received in the course	9 (8–10)	10 (9–10)	8.5 (8–10)	0.17
Your satisfaction to the overall course	10 (9–10)	10 (9–10)	9 (9–10)	0.44
How do you recommend incorporate the online course into the conventional offline intern training program in the future after the pandemic	10 (8–10)	10 (9–10)	10 (9.2–10)	0.30

^a^The three groups were compared using Kruskal–Wallis Test.

When the 3-week course was completed, a web-based anonymous survey was conducted using online questionnaire to get feedback from the interns. All the 92 interns (N = 92) engaged in our online education program responded to this survey on a voluntary basis (response rate = 100%). The survey covered questions about the interns’ satisfaction to our online course (i.e., satisfaction to the curriculum framework and its logic, satisfaction to the practicality of knowledge they obtained, satisfaction to the new knowledge and concepts they captured, and satisfaction to the overall course) and how they recommend the incorporation of the online course into the future intern training mode after the crisis (Table 1). A 10-point scale was adopted in the survey with 1–2 = ‘very dissatisfied’ or ‘strongly not recommend’, 3–5 = ‘dissatisfied’ or ‘not recommend’, 6–8 = ‘satisfied’ or ‘recommend’, 9–10 = ‘very satisfied’ or ‘strongly recommend’. A higher score corresponds to a higher satisfaction or intention to recommend. Besides, the interns’ opinions about the course contents, including new patients’ admission, videos of ward rounds and typical clinical cases, videos of discussions on difficult clinical cases, interactive case discussions and analyses, medical record writing (by the interns) and feedback, practice training and feedback, and didactic lectures, were also collected. The final scores of all the interns in the 3 groups were collected and compared.

### Statistical analysis

The counting data was described by percentage, while the descriptive data were expressed as median and interquartile range. Data processing and statistical analysis were performed using the SPSS program (version 22.0). Since the data did not conform to a normal distribution, the analysis of inter-group differences was performed by non-parametric test (Kruskal–Wallis Test or Mann–Whitney U Test). P < 0.05 was considered as statistically significant.

## Results

**Figure 1.**
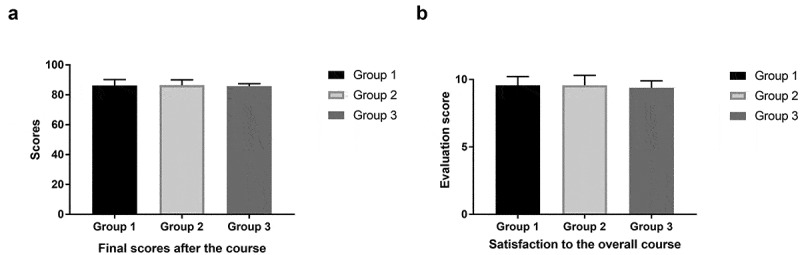
Comparison of the final scores and the satisfaction to the overall course among the 3 groups of interns. (a) No significant difference was detected in their final scores. (b) No significant difference was detected in their satisfaction to the overall course. Group 1 were students in their 5th year of a 5-year clinical medicine program, Group 2 were students in their 6th or 7th year of an 8-year clinical medicine program, Group 3 were students in their 5th year of a 5-year psychiatry program

**Figure 2.**
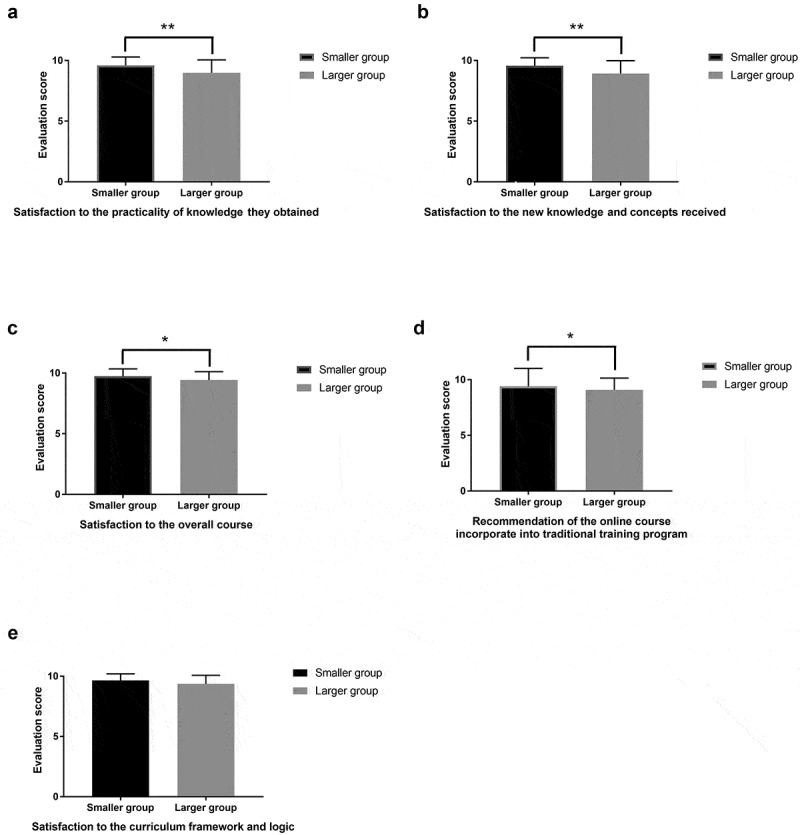
Comparison of the evaluation results from interns engaged in a smaller group and in a larger group. (a) Interns in a smaller group rated higher in the ‘satisfaction to the practicality of knowledge they obtained’. (b) Interns in a smaller group rated higher in the ‘satisfaction to the new knowledge and concepts received’. (c) Interns in a smaller group rated higher in the ‘satisfaction to the overall course’. (d) Interns in a smaller group rated higher in the ‘recommendation of the online course incorporate into traditional training program’. (e) Interns in a smaller group rated marginally higher in the ‘satisfaction to the curriculum framework and logic’. * p < 0.05, ** p < 0.01, and *** p < 0.001

The differences between the 3 groups concerning the final scores were compared using Kruskal–Wallis Test. No significant difference was detected between the final scores of different groups of interns (p = 0.676 > 0.05) (Figure 1), indicating that the interns were able to achieve similar outcomes after the course, despite the different medical educational programs they were in. For the satisfaction to the curriculum framework and its logic, 91% (84 of 92) of the interns reported ‘very satisfied’, 9% (8 of 92) reported ‘satisfied’, and 0% reported ‘dissatisfied’ or ‘very dissatisfied’. The median score was 10 (interquartile range, 9–10). For the satisfaction to the practicality of knowledge they obtained, 76% (70 of 92) rated ‘very satisfied’, 24% (22 of 92) rated ‘satisfied’, and 0% rated ‘dissatisfied’ or ‘very dissatisfied’. The median score was 10 (interquartile range, 9–10). For the satisfaction to the new knowledge and concepts they captured, 74% (68 of 92) reported ‘very satisfied’, and the other 26% (24 of 92) reported ‘satisfied’. The median score was 9.5 (interquartile range, 8–10). For the satisfaction to the overall course content, 90% (83 of 92) of the interns rated ‘very satisfied’, and the other 10% (9 of 92) rated ‘satisfied’. The median score was 10 (interquartile range, 9–10). For the intention to recommend incorporating the online course into the conventional offline intern training program after the pandemic, 78% (72 of 92) reported ‘strongly recommend’, 21% (19 of 92) reported ‘recommend’, and only 1 respondent reported ‘strongly not recommend’ (1%, 1 of 92). The median score was 10 (interquartile range, 9–10). As it can be seen, the overall online program achieved the highest rating. Concerning all the items above, there was no significant difference between the ratings from different groups of interns (p > 0.05) (Table 1 and Figure 1). Moreover, the course evaluation scores did not change significantly when comparing the first 3 batches of interns with the last 3 batches using the Mann–Whitney U Test (p > 0.05). Notably, the interns from a batch of a smaller group size (<15 students) were likely to give a significantly higher rating than those from a batch of a larger group size (≥15 students). More specifically, their ratings were higher in the ‘satisfaction to the practicality of knowledge they obtained’ (p = 0.003 < 0.05), ‘satisfaction to the new knowledge and concepts they captured’ (p = 0.003 < 0.05), ‘satisfaction to the overall course’ (p = 0.011 < 0.05), and ‘recommendation of incorporating the online course into the conventional training program’ (p = 0.012 < 0.05), and were marginally higher in the ‘satisfaction to the curriculum framework and its logic’ (p = 0.051) (Figure 2). The results indicated that the interns might have a better learning experience with a smaller learning group size, which may be due to the fact that the faculty members can spend more time on each student so as to meet their individual needs when the class size is small.

**Figure 3.**
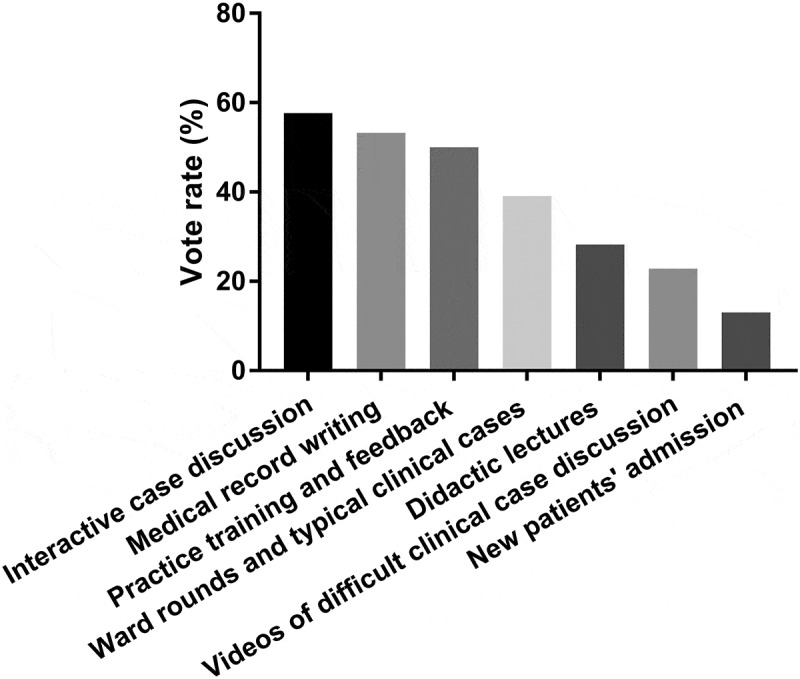
Interns’ preference to the contents of the course

The interns’ opinions about what content of the course was most helpful and useful were also summarized (multiple choices). Specifically, interactive case discussion and analysis received the highest rating, i.e., 58% (53 of 92), followed by medical record writing (53%, 49 of 92), practice training and feedback (50%, 46 of 92), videos of ward rounds and typical clinical cases (39%, 36 of 92), didactic lectures (28%, 26 of 92), videos of difficult clinical case discussion (23%, 21 of 92), and new patients’ admission (13%, 12 of 92) (Figure 3). The results suggested that the interns had a greater interest in interactive training as well as classes that they can get practice and feedback from. Furthermore, they preferred watching ward rounds in clinical settings and listening to analyses of clinical cases by their attending physicians at the bedside, rather than attending didactic lectures. Suggestions from the interns on course improvement included increasing the proportion of interactive discussions, videos of ward rounds and analyses of typical clinical cases, tracing the everyday treatment of clinical cases, and integrating the online course with offline training.

## Discussion

Our results were consistent with several other recently conducted studies concerning the good teaching effects of online courses [3,7]. Particularly, our investigation supported that the online program works effectively for clinical interns in the department of neurology, where the significance of conventional offline training is often deemed unshakeable. According to our survey, our online course received consistent positive recognition from the interns, and almost all the students (99%, 91 of 92) recommended popularizing the online course after the crisis. The unique advantages and positive outcomes of online medical training were neglected to a large extent in the past, while our finding implied that online training is worth to be promoted in the future as a complement to the face-to-face practical hospital training which is still irreplaceable. It is noteworthy that, despite the advantages of online training, the interns must still get involved in clinical practice such as examining real patients, operating lumbar puncture for real patients under supervision, and dealing with unexpectable clinical conditions, in order to acquire hands-on experience. Therefore, offline clinical training still plays an essential and irreplaceable role in the neurology intern training and should be recovered as soon as possible once the COVID-19 pandemic situation is under control.

Our results also indicated that a smaller online learning group/class size (<15 students) might exert a better effect than a larger group/class size. Even though the online learning mode allows a number of users to attend the course concurrently, the quality of the course and the individual experience of each student may be affected by the group size. We divided the interns into 3 groups based on their medical educational programs and grades, for the purpose to see whether our online training course can cater for students from different programs and with different knowledge sets. As a result, no significant difference was detected between different groups concerning the final scores and course evaluation, which suggested that the interns were able to achieve similar learning outcomes regardless of the medical educational program they were in and that our course can be promoted in a large student base. Regarding the training content, the interns generally preferred interactive discussions as well as classes they can get practice and feedback from such as medical record writing and practical training of simulated physical examination, CPR and lumbar puncture. This is probably because that they can notice their own problems in the discussions and practice, thereby achieving a better progress. Although face-to-face clinical training was infeasible at the moment, the interns appreciated the opportunity to practice in a simulated situation and get feedback from experienced clinicians. In addition, they preferred the combination of clinical case analysis and theoretic teaching rather than didactic lectures alone, suggesting that a more lively and clinical case-based course is more popular and effective in stimulating the medical interns’ learning interest and motivation.

SPOC can overcome some limitations of MOOC and the conventional didactic lecture-based learning mode and therefore has a promising prospect [11]. Teaching based on the blending learning mode can better meet the needs of students, and enhance their critical thinking and learning efficiency [13,15]. The advantages of online training can partially compensate for the shortcomings of offline clinical training. First, online teaching can provide all students with typical clinical case analyses and lectures by high-level clinical teachers (even by teachers from overseas) through video recording, which helps guarantee the teaching quality. Besides, rare cases (such as a patient with Charcot-Marie-Tooth Disease) can also be shared among all the students. Second, online classes allow for interactive discussion of clinical cases, which is useful for training the interns’ clinical reasoning and overall analytical ability. Third, diverse and blending learning methods can improve the motivation and initiative of the students. Finally, modern technology has made online learning easily available at a low cost, and online classes can be attended repeatedly without the limitation of geographic location [21].

Except for the advantages of online training itself, our course also addressed the particularities of neurology training, which is a highly rated aspect by the interns. First, unlike some other departments where most patients are diagnosed definitely, the interns in the neurology clerkship must learn how to comprehensively consider subjective symptoms, clinical signs, auxiliary examinations and a complete history to arrive at a most likely diagnosis, to suggest other necessary examinations, and to make an optimal treatment plan. Moreover, the treatment should be dynamically adjusted according to the tolerance and treatment response of the patient. All the procedures above were presented to the students in our online training course through video recording, PowerPoint, and interactive discussion. Second, more training of critical thinking is needed during the neurology clerkship compared to other disciplines since many neurological disorders present similar clinical features and radiographic findings. Such training was realized in our online course by showing videos of difficult clinical case discussions and organizing interactive case discussions. Third, in the neurology department, it is common that some cases require a long time (even over a month) to identify the cause and proper treatment. However, due to a short rotation period (2–3 weeks) that the interns can stay in the department, it is difficult for them to follow those cases. Online course can overcome this problem by collecting complete data beforehand. Last but not least, the hands-on skills required to be mastered during the neurology clerkship can be practiced more easily at home than at surgical clerkship settings.

Nevertheless, compared to conventional clinical training in the hospitals, the students are in face of new challenges during attending the online learning program, such as more stringent requirements on self-discipline and autonomous learning. For the faculty, the demand for online teaching opens new opportunities for them to receive relevant training and to create useful online modules for their courses [22]. The online clinical intern training model based on SPOC has also been applied in other departments (including internal medicine and surgery) at our hospital in 2020. Despite some differences in course arrangement, the online training in other departments obtained positive recognition from the interns as well, especially in internal medicine departments (such as Cardiology and Endocrinology), which further evidenced the application value and prospect of the remote intern training model. The experience can help set an online education module to minimize the disruptions to the clinical training of medical students in case of similar emergency situations in the future, and can also help prompt medical schools and hospitals to permanently incorporate distance teaching into their conventional intern training curricula as a supplement in order to enhance the flexibility of health professional education and optimize the training effect [23–25].

### Limitations

First of all, our findings are limited by the implementation of the study at a single medical center. Other limitations include small sample size of the intern cohort from the program of psychiatry, limited number of questions in the theoretical examination, and lack of a control group that attended both online and offline training courses. We hope that our experience can serve as a reference for medical schools and hospitals in China and other countries. However, since the pandemic is an emergency, further studies such as comparisons between the conventional training program, the online program, and the combination of both are still needed after the crisis.

## Conclusions

Overall, our online clinical training program based on SPOC and blending learning mode achieved a good teaching effect and high ratings from interns of distinct medical educational programs in the department of neurology, which has a good reference effect. Specifically, the results suggested that a smaller learning group/class size (<15 students) may exert a better effect than a larger group/class size. In addition, the interns prefer interactive discussions and classes that they can get practice and feedback from, rather than video watching and didactic lectures. The online clinical neurology training course based on SPOC and blending learning mode is worthy of popularization and incorporation into the conventional intern training mode in the future for the cultivation of high-level medical talents, even though further studies are still needed since the pandemic is an emergency situation.

## Supplementary Material

Supplemental Material
