# Supplementary material for: Remote clinical training practice in the neurology internship during the COVID-19 pandemic
Source: Med Educ Online. 2021 Mar 8;26(1):1899642. doi: 10.1080/10872981.2021.1899642 (PMC7946031; doi:10.1080/10872981.2021.1899642)
Supplement: Supplemental Material [file ZMEO_A_1899642_SM6211.docx]

**Supplemental Table 1.** The curriculum of our online course.

| Week | Day | Contents | Forms |
| --- | --- | --- | --- |
| 1 | 1 | Physical examination of the nervous system (1) | Didactic lecture, recorded video of physical examination of real patients, relevant homework |
|  | 2 | Physical examination of the nervous system and videos of difficult case discussions (2) | Didactic lecture, recorded videos of physical examination and difficult case discussions of real patients, relevant homework |
|  | 3 | Medical history taking and medical record writing of patients with neurologic disorders | Didactic lecture, real clinical case analysis, relevant homework |
|  | 4 | New patients’ admission (1) | Recorded videos of real patient encounter including medical history taking and physical examination, homework of medical record writing by the interns |
|  | 5 | New patients’ admission (2) | Recorded videos of real patient encounter including medical history taking and physical examination, homework of medical record writing by the interns |
| Self-study during the weekend | | | |
| 2 | 1 | Ward rounds and analysis of typical clinical cases at bedside (1) | Recorded or live videos of ward rounds and typical clinical case analysis by the attending physicians, home work of clinical case analysis |
|  | 2 | Feedback of the homework of medical record writing, interactive clinical case discussions and analysis (1) | Interactive discussions with guidance from the instructors, relevant homework |
|  | 3 | Ward rounds and analysis of typical clinical cases at bedside (2) | Recorded or live videos of ward rounds and typical clinical case analysis by the attending physicians, relevant homework |
|  | 4 | Practice training of physical examination, cardiopulmonary resuscitation and lumbar puncture | Recorded or live videos of practice in the clinical setting and explanation by the instructors, homework of recording videos of practice at home |
|  | 5 | Ward rounds and analysis of typical clinical cases at bedside (3) | Recorded or live videos of ward rounds and typical clinical case analysis by the attending physicians, didactic lecture of neurologic disorders, relevant home work |
| Self-study during the weekend | | | |
| 3 | 1 | Ward rounds and analysis of typical clinical cases at bedside (4) | Recorded or live videos of ward rounds and typical clinical case analysis by the attending physicians, didactic lecture of neurologic disorders, home work of clinical case analysis |
|  | 2 | Feedback of video recording of home practice, interactive clinical case discussions and analysis (2) | Interactive discussions with guidance from the instructors, relevant homework |
|  | 3 | Ward rounds and analysis of typical clinical cases at bedside, videos of difficult case discussions (5) | Recorded or live videos of ward rounds and typical clinical case analysis by the attending physicians, didactic lecture of neurologic disorders, videos of difficult case discussions, home work of clinical case analysis |
|  | 4 | Interactive clinical case discussions and analysis (3) | Interactive discussions with guidance from the instructors, relevant homework |
|  | 5 | Theoretical examination |  |
| Self-study during the weekend | | | |
